# Exploring the *Arabidopsis* Proteome: Influence of Protein Solubilization Buffers on Proteome Coverage

**DOI:** 10.3390/ijms16010857

**Published:** 2014-12-31

**Authors:** Claudius Marondedze, Aloysius Wong, Arnoud Groen, Natalia Serrano, Boris Jankovic, Kathryn Lilley, Christoph Gehring, Ludivine Thomas

**Affiliations:** 1 Biological and Environmental Sciences and Engineering Division, 4700 King Abdullah University of Science and Technology, Thuwal 23955-6900, Saudi Arabia; E-Mails: claudius.marondedze@kaust.edu.sa (C.M.); aloysius.wong@kaust.edu.sa (A.W.); natalia.serrano@kaust.edu.sa (N.S.); boris.jankovic@kaust.edu.sa (B.J.); christoph.gehring@kaust.edu.sa (C.G.); 2 Cambridge Centre for Proteomics, Cambridge System Biology, University of Cambridge, Department of Biochemistry, Tennis Court Road, Cambridge CB2 1QR, UK; E-Mails: ajg86@cam.ac.uk (A.G.); ksl23@cam.ac.uk (K.L.)

**Keywords:** two-dimensional gel electrophoresis (2DE), *Arabidopsis thaliana*, detergent, mass spectrometry (MS), OFFGEL fractionation

## Abstract

The study of proteomes provides new insights into stimulus-specific responses of protein synthesis and turnover, and the role of post-translational modifications at the systems level. Due to the diverse chemical nature of proteins and shortcomings in the analytical techniques used in their study, only a partial display of the proteome is achieved in any study, and this holds particularly true for plant proteomes. Here we show that different solubilization and separation methods have profound effects on the resulting proteome. In particular, we observed that the type of detergents employed in the solubilization buffer preferentially enriches proteins in different functional categories. These include proteins with a role in signaling, transport, response to temperature stimuli and metabolism. This data may offer a functional bias on comparative analysis studies. In order to obtain a broader coverage, we propose a two-step solubilization protocol with first a detergent-free buffer and then a second step utilizing a combination of two detergents to solubilize proteins.

## 1. Introduction

To date, a number of different protein extraction methods and solubilization buffers have been applied in plant proteomics studies and they differ from those typically used in prokaryote and animal studies [1]. For proteomics analyses, standardized sample preparation that ensures consistent results is crucial in order to obtain high-quality resolution and greater coverage of the proteome [2] and allows the comparison of data from different studies. For example, proteins from apple (*Malus domestica*), avocado (*Persea americana*), banana (*Musa americana*) and orange (*Citrus* × *sinensis*) fruits extracted in a study using either a phenol/chloroform or a trichloroacetic acid (TCA) in acetone protocol yielded different proteome profiles [3,4]. Furthermore, plant proteomics presents difficulties caused by the structural characteristics of plant tissues including the cell wall matrix [3,5,6,7,8] and secondary metabolites that can cause problems in two-dimensional gel electrophoresis (2DE) and mass spectrometry (MS) [9].

Although 2DE is a valuable technique used in plant proteomics, a lack of resolution notably for hydrophobic-membrane proteins and basic proteins, the presence of multiple proteins in a single spot, and a limited dynamic range remain obstacles in profiling whole plant proteomes. The alternative gel-free approaches including protein antibody arrays [10] and liquid chromatography coupled to tandem mass spectrometry (LC–MS/MS) [11] can overcome some of the limitations of 2DE. The LC–MS/MS technique is however incompatible with most detergents required for solubilizing hydrophobic proteins. Variation in the solubilized proteome also depends on protein precipitation methods often employed, which includes ammonium acetate in methanol [7,12,13], ethanol [14], acetone, methanol, TCA [13,14,15] and a combination of TCA and acetone [15,16]. The latter two techniques also inhibit unwanted proteolytic activity of the sample during precipitation [17], while limiting interference with secondary metabolites [18,19]. Moreover, the combination of precipitation method and solubilization buffer can also cause differences in protein recovery and resulting proteomes [3,4,6,15]. Recent studies have started assessing the effects of detergent in solubilization on proteome coverage from *E. coli* [20] and biofilm-forming bacteria [21]. To the best of our knowledge, however no such studies has been performed in plants.

The aim of the present study is to give a critical account of the effects of detergent-specific protein species in cell suspension cultures in the model plant *Arabidopsis thaliana*, an experimental system with reduced levels of Rubisco that notoriously masks low abundance proteins in studies utilizing photosynthetic plant material. Here, we concentrate on comparatively assessing the influence of different solubilization buffer systems containing either a single-step solubilization process with a buffer containing one detergent (sodium dodecyl sulfate (SDS), Triton-X-100 (TRIT), 3-[(3-Cholamidopropyl)dimethylammonio]-1-propanesulfonate (CHAPS) or non-detergent sulfobetaine 201 (NDSB)) or no detergent (ND), or a two-step solubilization process, which consists in, first, protein solubilization in ND-based buffer and then a second solubilization step in a urea-thiourea lysis buffer combining SDS and NDSB for the recovery of proteins. We employ protein level separation using 2DE or OFFGEL fractionation at peptide level to investigate and compare the proteome maps resulting from using these different buffer systems. In addition, we characterize the effects on the buffer-specific proteomes by analyzing localization, hydrophobicity, number of predicted membrane domains, and pattern of post-translational modifications (PTMs).

## 2. Results and Discussion

Proteins were extracted from Arabidopsis cell suspension cultures using TCA in acetone precipitation followed by a single-step solubilization in a urea-thiourea lysis buffer containing ND or either CHAPS, NDSB, SDS, TRIT, or a two-step solubilization process. Solubilized proteins were analyzed either by 2DE or OFFGEL fractionation followed by LC–MS/MS (Figure S1).

### 2.1. Proteome Analyses

To gain insight into the visual differences of the proteomes solubilized by the five buffers tested, protein extracts were resolved by 2DE. The 2DE analysis revealed substantial qualitative differences among the five buffer systems tested for the single solubilization process, which can be attributed to their ability to solubilize proteins. The CHAPS-containing buffer system allowed for the visualization of a greater number of protein spots, with >1200 spots resolved on 2DE gels on average (Table 1). On average, the ND-based buffer led to gel images with about 1200 spots, the second highest number of detected protein spots, while the lowest spot number was visualized with TRIT (1072 spots). The analysis also revealed that 36 and 75 spots were either specifically present or absent in only one buffer system (Figure S2). Of the 36 protein spots, 13 were detected with ND-based buffer, 12 with CHAPS, 10 with TRIT and one with SDS, whereas NDSB did not result in any unique spots. Only these unique spots were processed for identification by LC–MS/MS (Table S1) and six remained unidentified (spots 19, 418, 477, 492, 562, 1295). These results suggest that maximum coverage cannot be achieved using just one buffer system. Detection of ND-specific spots reveals that some proteins are soluble only and subsequently resolved in the absence of detergent. However, a detergent-free buffer may result in the under representation of hydrophobic proteins, such as membrane proteins. It is important to note that the focus of this study is on the qualitative differences of different solubilization buffers on the extracted proteome profile and coverage, and not the entire proteome visualized. Some of the other non-identified protein spots might represent isoforms of the identified proteins that might have resolved at different isoelectric points and/or molecular weights, thus, leading to inaccurate quantitative estimations. Although 2DE is a versatile and informative tool for comparative proteomics, hydrophobic proteins are rarely detectable with this technique [22,23], especially those with positive grand average of hydropathy (GRAVY) indices [24]. In agreement with this, only four proteins, corresponding to five spots, obtained a positive GRAVY score, and three were predicted to have transmembrane (TMs) domains (Table S1).

**Table 1:** Number of proteins identified from the different solubilization buffers for two-dimensional gel electrophoresis (2DE) and OFFGEL fractionation methods. Standard errors from three biological replicates are shown for 2DE analysis (±).

Number Identified Proteins	NDSB	CHAPS	ND	SDS	TRIT
**Average 2DE spots per gel**	1187 ± 21	1277 ± 41	1267 ± 22	1238 ± 17	1072 ± 29
**OFFGEL fractionated samples**	3383	3092	2906	3058	2990

**Figure 1:**
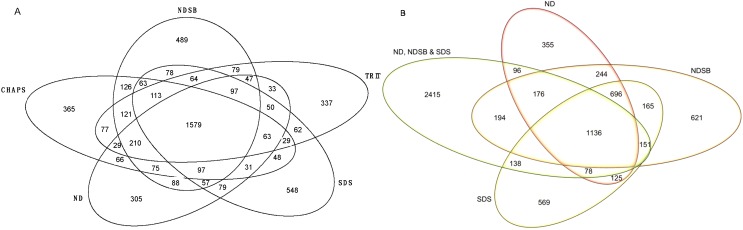
Venn diagrams of the number of *Arabidopsis* proteins identified using different buffer systems after OFFGEL fractionation. The lysis buffer containing 7 M urea and 2 M thiourea was supplemented with either no detergent (ND), 4% (*w*/*v*) 3-[(3-Cholamidopropyl)dimethylammonio]-1-propanesulfonate (CHAPS), 4% (*w*/*v*) non detergent sulfobetaine 201 (NDSB), 2% (*v*/*v*) Triton X-100 (TRIT), 1% (*w*/*v*) sodium dodecyl sulfate (SDS) or a two-step solubilization process consisting first in ND-based buffer and then a combination of 4% (*w*/*v*) NDSB and 1% (*w*/*v*) SDS (*i.e.*, ND, NDSB and SDS) to solubilize total protein extracts from Arabidopsis cell suspension cultures. After trypsin digestion, resulting peptides were OFFGEL fractionated, desalted and analyzed by LC–MS/MS. Proteins were identified using MASCOT search engine. Venn diagrams show number of protein identifications for (**A**) each of the buffer systems tested and overlaps among the five individual buffer systems and (**B**) the two-step solubilization process, ND-, followed by NDSB and SDS-based buffers.

Combining OFFGEL fractionation to LC–MS/MS analysis allowed for a total of 5505 unique proteins to be positively identified. The NDSB-based buffer resulted in the largest protein identification with a single buffer (3383 proteins; Figure 1), although no unique protein spots were detected at 2DE level with this particular buffer. With CHAPS, 3092 proteins were identified, 3058 with SDS, 2990 with TRIT and 2906 with ND (Table 1). Of the total proteins identified, 28.7% (1579 proteins) were common to all buffer systems, and 18% (548), 14% (489), 12% (365), 11% (337) and 10.5% (305) were unique to SDS-, NDSB-, CHAPS-, TRIT-, and ND-containing buffers, respectively (Figure 1A). Additionally, some proteins were present in at least two buffer systems. Using the two-step solubilization process, a greater amount of proteins (4384) were identified of which, 1784 were detected only after the first solubilization in ND-based buffer and 548 after the second solubilization in SDS- and NDSB-based buffer. A total of 1136 proteins were common to ND-, NSDB-, and SDS-based buffers (Figure 1B). Interestingly, 2415 proteins (55%) were only identified after the use of the two-step solubilization buffer and not in any other buffer system alone. This clearly highlights the benefit from the two-step solubilization process over the single-step one. It is however important to note that a number of proteins were still specifically identified after using the ND-, NDSB-, and SDS-based buffer alone, as 355, 621 and 569 proteins, respectively, were identified with these buffer systems only.

### 2.2. Functional Enrichment of Displayed Proteomes

**Figure 2:**
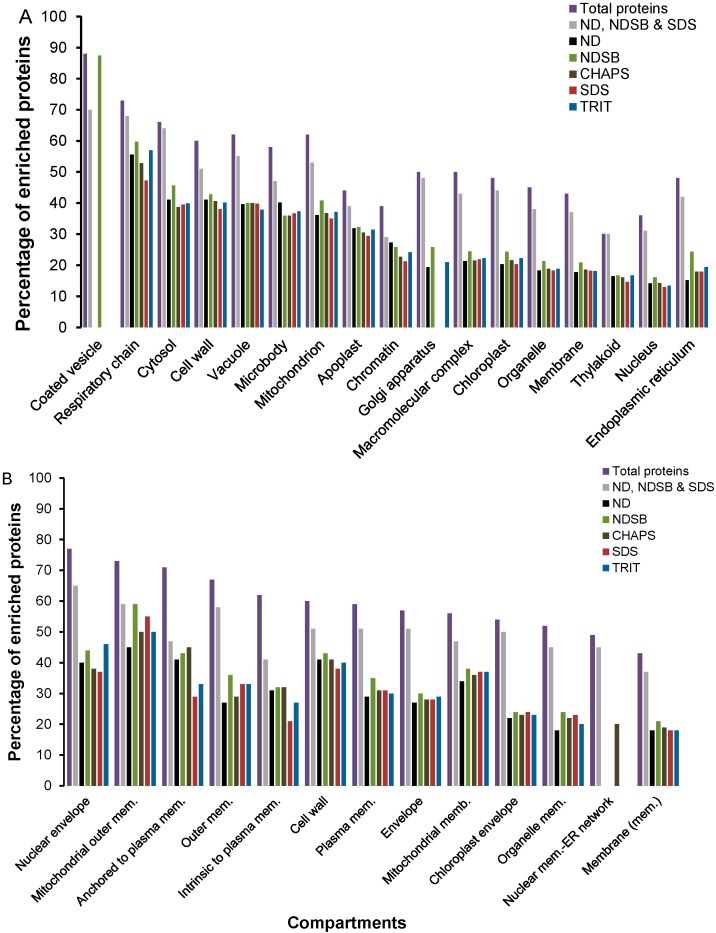
Enriched cellular compartments in (**A**) total unique and (**B**) membrane-associated proteins identified in the five buffer systems. Gene ontology (GO) analyses were performed using the entire dataset of identified proteins and then using the buffer-specific datasets separately. Cellular compartments that were detected as enriched are shown in the graph (*p* ≤ 0.05 and false discovery rate (FDR) ≤ 1%). Bars represent the ratio of enrichment against the total number of proteins for each category.

Proteins identified after OFFGEL fractionation showed a bias towards some organelles and cellular compartments, particularly “coated vesicle”, which was enriched only in NDSB-containing buffer, followed by “respiratory chain” and “cytosol” (Figure 2A). Enrichment of the latter was expected as close to 25% of all experimentally localized proteins in the SUBcellular *Arabidopsis* database (SUBA) are cytosolic [25]. However, since the cytosol is an aqueous environment, a large portion of its proteins is hydrophilic, suggesting enrichment for hydrophilic proteins, particularly with ND- and NDSB-containing buffers. Enrichment for “Golgi apparatus” was only detected in ND-, NDSB-, and TRIT-containing buffers. Other categories showed similar levels of enrichment in the five buffer systems, with the exception of “mitochondrion” and “endoplasmic reticulum” (ER), which showed higher enrichment in NDSB-based buffer, “respiratory chain” in NDSB- and TRIT-containing buffers and “Golgi apparatus” that was not detected in CHAPS- and SDS-based buffers. In the two-step solubilization approach, all categories were represented and importantly, these categories showed greater enrichment than the respective ND-, NDSB-, and SDS-based buffers (Figure 2). Considering specifically the “small guanosine triphosphatase (GTPase)” (19 proteins), “cell surface receptor” (32 proteins) and “Golgi vesicle transport” (11 proteins) categories, unique isoforms were identified with different buffer systems (Table S2). The NDSB-containing buffer retrieved more proteins from each of the three categories while no protein from the “Golgi vesicle transport” category was detected with ND.

Membrane-associated categories were also enriched (Figure 2B), particularly after the two-step solubilization process. The SDS-based buffer showed a lower enrichment in proteins from the “anchored to plasma membrane” and “intrinsic to plasma membrane” categories, while proteins from the “nuclear membrane-ER network” were only enriched in CHAPS-based buffer and in the two-step solubilization process. Functional annotations in terms of biological processes, molecular function and localization of all the identified proteins by LC–MS/MS are shown in Table S2. Overall, these observations clearly indicate that a single solubilization buffer is unable to capture the full complementary of the proteome and a cocktail of complimentary detergents (such as NDSB and SDS) can achieve a better qualitative representation and proteome coverage.

### 2.3. Physico-Chemical Properties of Buffer-Specific Proteins

**Figure 3:**
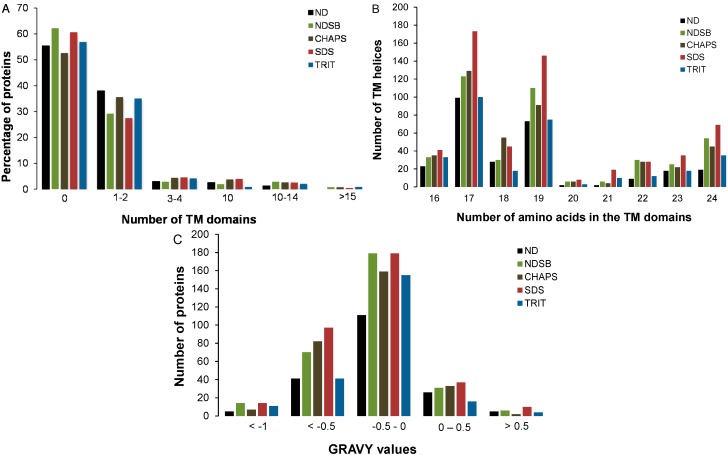
Number of proteins containing (**A**) transmembrane domains, (**B**) number of amino acids per domain and (**C**) grand average of hydropathy (GRAVY) index. (**A**) Transmembrane (TM) domains were computationally predicted with the Hidden Markov Model for TOPology Prediction (HMMTOP) tool in the proteins identified in a single buffer system only in relation to all buffer-specific proteins identified; (**B**) Number of TM helices detected in increasingly long predicted TM domains from the proteins identified in a single buffer system only was calculated on the basis of HMMTOP predictions; (**C**) GRAVY index was predicted using the GRAVY calculator for all proteins that were specifically identified in a single buffer system. Positive scores define proteins as hydrophobic, while negative scores classify proteins as hydrophilic.

A prediction of TM domains and analysis of GRAVY after OFFGEL fractionation showed that in the single-step buffer system, each buffer enabled the solubilization of a subgroup of proteins with certain physico-chemical properties. Close to 40% of proteins solubilized only in ND-, CHAPS-, or TRIT-based buffers contained one or two TM domains, while for NDSB and SDS this was only 30% (Figure 3A). Only a small amount of proteins were predicted to contain ≥3 TM domains irrespective of the buffer used. Comparison between the number of amino acids and the number of helices in TM domains showed that more TM helices were detected in 17 and 19 amino acid-long TM domains, and this was particularly evident with the SDS-based buffer (Figure 3B). The detection of a greater number of TM helices with this buffer system might also be due to the fact it identified the highest number of buffer-specific proteins (548 proteins; Figure 1).

The GRAVY values, which estimate protein hydrophobicity, of buffer-specific proteins ranged from −1.91 to 0.82, revealing that most proteins solubilized in a single-buffer system were slightly hydrophilic (GRAVY from −1 to 0; Figure 3C), and this was more prominent in the NDSB- and SDS-based buffers. A limited number of proteins were either highly hydrophilic (GRAVY ≤ −0.5), or hydrophobic (positive GRAVY). An increased hydrophobic protein recovery was achieved with the SDS-based buffer and this was not unusual since the ionic SDS detergent is known to be a highly efficient solubilizer of hydrophobic proteins [12]. This buffer also allowed for the detection of a greater number of proteins harboring TM domains and palmitoylation sites.

### 2.4. Predictions of Post-Translational Modifications

**Figure 4:**
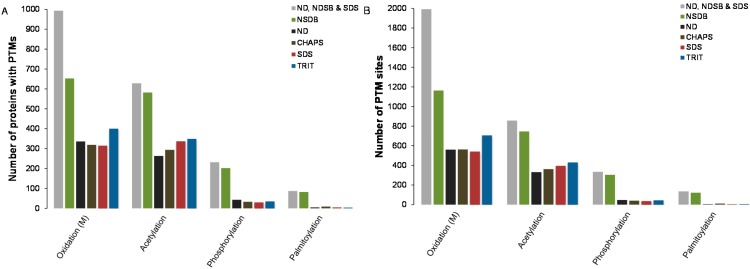
Number of predicted (**A**) post-translation modifications (PTMs) and (**B**) sites for individual buffer systems and the two-step solubilization process. During protein identification in the MASCOT and Scaffold, acetylation, oxidation, phosphorylation and palmitoylation were set as variable modifications. Bars represent the (**A**) number of proteins and (**B**) PTM sites that contain the PTM of interest.

Looking at the entire set of identified proteins from each buffer system tested after OFFGEL fractionation, acetylated, oxidized, phosphorylated and palmitoylated sites were detected by MASCOT and Scaffold (*p* ≥ 95%; Figure 4A,B). Greater numbers of these four PTMs were predicted after the two-step solubilization process and NDSB-containing buffer, with 856 and 746 acetylated sites in 628 and 582 proteins, 1992 and 1163 oxidized sites in 993 and 653 proteins, 335 and 304 phosphosites in 232 and 202 phosphorylated proteins and 135 and 121 palmitoylated sites located in 87 and 82 proteins, respectively. Detection of phosphorylated and palmitoylated protein sites was limited in all samples, but was higher with the two-step solubilization process and NDSB-containing buffer (Figure 4B). Predictions of other PTMs were carried out using the Big-PI plant predictor for glycosylphosphatidylinositol (GPI) site detection, and this suggested that most proteins containing GPI modifications also possessed palmitoylation sites (Table S3). Furthermore, myristoylated sites were predicted with the myristoylation (MYR) predictor tool locating 100 MYR signals of which, 16 contained GPI sites. Interestingly, 14 of these 16 predicted myristoylated proteins also contained palmitoylation sites (Table S4). Taken together and based on these selected PTMs, the data implies that the two-step solubilization process allowed for the detection of the highest number of predicted PTMs, suggesting that this combination of buffers may be better suited for PTM-based proteomic studies. Interestingly, for all four PTMs considered, sites of modification outnumbered the number of proteins, indicating that the recovery of multiple modified proteins was achieved.

## 3. Experimental Section

### 3.1. Arabidopsis Cell Suspension Culture and Protein Extraction

Cells derived from *Arabidopsis thaliana* (ecotype Columbia 0) roots were grown in 100 mL Gamborg’s B-5 [26] basal salt mixture (Sigma-Aldrich, St. Louis, MO, USA) with 2,4-dichlorophenoxyacetic acid (2,4-D; 1 mg·mL^−1^) and kinetin (0.05 μg·mL^−1^) in 250 mL sterile flasks. Cells were grown in a growth chamber (Innova^®^ 43, New Brunswick Scientific Co., Edison, NJ, USA) with shaking at 120 rpm, under photosynthetic light with 12 h light/12 h dark cycles at 23 °C and subcultured every 10 days. At 10 days-post subculturing, medium was drained off using Stericup^®^ filter unit (Millipore, Billerica, MA, USA), and cells were immediately frozen in liquid nitrogen and stored at −80 °C until use.

Approximately 1 g of frozen Arabidopsis cells was homogenized in 10 volumes of ice-cold 10% (*w*/*v*) TCA in acetone using a PowerGen 125 grinder (Fisher Scientific, Rockford, IL, USA) and incubated overnight at −20 °C. Proteins were pelleted at 3900× *g* for 15 min at 4 °C, washed three times in 80% (*v*/*v*) ice-cold acetone, solubilized in equal volume of 7 M urea, 2 M thiourea containing either ND, 4% (*w*/*v*) CHAPS, 1% (*w*/*v*) SDS, 2% (*v*/*v*) TRIT or 4% (*w*/*v*) NDSB and centrifuged at 20,800× *g* for 15 min. For the two-step solubilization process, the protein pellet obtained after TCA in acetone precipitation was first solubilized in ND-based buffer. The residual pellet after centrifugation was solubilized in a buffer containing 7 M urea, 2 M thiourea, 1% (*w*/*v*) SDS and 4% (*w*/*v*) NDSB. The two protein fractions were analyzed separately. Protein concentration was estimated by Bradford [27] with Quick Start™ Bradford reagent (Bio-Rad, Hercules, CA, USA) and bovine serum albumin as standard.

### 3.2. Comparative Two-Dimensional Gel Electrophoresis (2DE) of Arabidopsis Proteomes and In-Gel Digestion

Approximately 50 μg of proteins was purified with 2D clean-up kit (GE Healthcare, Uppsala, Sweden), according to manufacturer’s recommendations, resuspended in 125 μL of rehydration solution (7 M urea, 2 M thiourea, 4% (*w*/*v*) CHAPS, 40 mM dithiothreitol (DTT), 0.5% (*v*/*v*) immobilized pH gradient (IPG) buffer pH range 4–7) and used to passively rehydrate 7-cm long IPG strips pH range 4–7 (GE Healthcare, Pittsburgh, PA, USA). Isoelectric focusing (IEF) and polyacrylamide gel electrophoresis (PAGE) were carried out as previously described [28]. Three biological gels for each buffer system stained with SYPRO^®^ Ruby stain (Molecular Probes, Eugene, OR, USA) were imaged with a Typhoon™ 9410 scanner (GE Healthcare). Gel images were analyzed with Delta 2D v4.2 (Decodon, Greifswald, Germany). Gels were warped with group warping strategy. Similar regions/spots were automatically searched between two images and warped using exact warp mode. All automatically detected vectors were manually confirmed using dual channel view. In case of improper or incomplete warping, extra vectors were manually added to ensure good overlay of the two images. Once all images were warped, a fusion image comprising of all spots detected was created, which was then used for automated spot detection with the union fusion algorithm. This algorithm retains all detected spots even if present in only one image. Artifacts were deleted; spots were edited by addition of non-detected spots, and splitting and/or joining spot clusters. Spot boundaries were automatically transferred back to the original images. Spot quantities were calculated by summing pixel intensities within the spot boundaries and used for analyzing gene expression. Normalized expression profile data was used to statistically assess variations in protein spot expression. Differentially expressed spots between two groups were calculated using Student’s *t*-test (*p* ≤ 0.05) and permutation-based method was used to avoid biased results that may arise within replicate gels if spot quantities are not normally distributed. Differentially expressed protein spots within at least two gels were calculated using one-way and two-way analysis of variance (ANOVA) (*p* ≤ 0.05). Adjusted Bonferroni correction was applied for false discovery rate (FDR) to control proportion of false positives and principal component analysis (PCA) was performed to determine samples/spots contributing most to the variance and their relatedness. Spots detected only in a particular buffer system were manually picked and *in-gel* trypsin digested, as described in [28].

### 3.3. Gel-Free Trypsin Digestion of Complex Protein Extracts

Approximately 1 mg of proteins was reduced with 5 mM DTT for 2 h at 37 °C and then alkylated with 14 mM iodoacetamide (IOA) for 30 min in the dark. Unreacted IOA was quenched with an extra 5 mM DTT for 15 min. Proteins were diluted to 2 M urea with 50 mM triethylammonium bicarbonate buffer (Sigma-Aldrich) and incubated at 1:50 ratio with trypsin (Promega, Madison, WI, USA) overnight at 37 °C. Peptides were desalted with Sep-Pak Vac tC18 cartridge (Waters, Milford, MA, USA), as described in [29] and purified using detergent removal columns, as recommended by the manufacturer (Pierce, Thermo-Scientific, Rockford, IL, USA), and fractionated with a 3100 OFFGEL fractionator (Agilent Technologies, Santa Clara, CA, USA).

### 3.4. Peptide Fractionation by OFFGEL Fractionator

Samples and 12-well IPG strips were prepared as recommended by the manufacturer and electrofocused to 20 kV at 20 °C. Peptides were fractionated by OFFGEL using 12-well IPG strip. Three biological samples for each buffer system were pooled and diluted to a final volume of 1.8 mL with 1.25× peptide OFFGEL stock solution (50% (*v*/*v*) glycerol solution, 10% (*v*/*v*) OFFGEL buffer pH range 3–10). Strips were rehydrated, as recommended by the manufacturer and electrofocused to 20 kV at 20 °C, at maximum 4500 V and 50 μA per strip, after 150 μL aliquots of sample was pipetted into each well. After focusing, 12 fractions per strip were separately collected; wells rinsed with 200 μL of 50% (*v*/*v*) acetonitrile (ACN) and 5% (*v*/*v*) formic acid (FA) twice for 15 min each and rinsing solution collected into their corresponding tubes and ACN was evaporated from the fractions using a Speed Vac concentrator (Thermo-Scientific, Rockford, IL, USA). The 12 collected fractions for each strip were purified with Sep-Pak Vac tC18, as described previously [29].

### 3.5. Protein Identification by LC–MS/MS

Peptides were resuspended in 5% (*v*/*v*) ACN and 0.1% (*v*/*v*) FA and analyzed with LTQ-Orbitrap Velos MS (Thermo-Scientific) coupled with a nanoelectrospray ion source (Proxeon Biosystems, Odense, Denmark), as described in [29]. Raw data were converted to mgf with Proteome Discover v1.2.0.208 (Thermo-Scientific) and submitted to a local MASCOT (Matrix Science, London, UK) server and searched against *Arabidopsis thaliana* in the *Arabidopsis* information resource (TAIR; release 10), with precursor mass tolerance of 10 ppm, fragment ion mass tolerance of ±0.5 Da, and strict trypsin specificity allowing up to one missed cleavage, carbamidomethyl modification on cysteine residues as fixed modification, and oxidation of methionine residues, phosphorylation of serine, threonine and tyrosine residues. MASCOT searches were then repeated with the same conditions except for the variable modifications, which were changed to palmitoylation on lysine and *N*-terminal acetylation as variable modifications. Proteins were considered positive if molecular weight search (MOWSE) score was ≥95% confidence limit (score ≥ 26). Data was further analyzed and validated with Scaffold v4.0.4 (Proteome Software, Portland, OR, USA) allowing for 0.1% FDR and Scaffold PTM v.

### 3.6. Computational Analysis of Identified Proteins

Identified proteins were considered for gene ontology cellular component, biological processes and molecular function enrichment analyses using Cytoscape v3.0.2 ([30]). Only categories with *p* values ≤0.05 and false discovery rate (FDR) ≤1% were considered (Table S3). Predictions for number and length of TM domains were achieved using the HMMTOP server [31,32]. GRAVY values were estimated with the GRAVY calculator [33]. Scaffold PTM (Proteome Software) that utilizes the Ascore algorithm [34] was used to identify and positioning of phosphorylation, oxidation, acetylation and palmitoylation sites at *p* ≥ 95%. Predictions of other PTMs were attempted using Big-PI Plant Predictor [35] for GPI, and the NMT–MYR predictor [36].

## 4. Conclusions

Firstly, the data presented in this study demonstrate that the five buffer systems tested yielded different proteomes, as visualized by 2DE analysis, each with a number of unique proteins. This suggests that no one buffer system is powerful enough to give a broad, let alone complete representation of the entire proteome. Secondly, the GO analysis revealed preferential enrichment of proteins from different cellular compartments and this can potentially influence the choice of detergent such as in the case of organelle proteomics. Thirdly, buffer systems also differently enrich candidate proteins with PTMs. Overall the detergent of choice for the solubilization of proteins is an important factor in obtaining a comprehensive view of the proteome. Since a single detergent solubilization system is insufficient for broad proteome coverage, we therefore proposed and demonstrated using the NDSB- and SDS-containing buffer systems, that a combination of complimentary detergents and/or a two-step solubilization protocol with first a detergent-free buffer and followed by the NDSB- and SDS-buffers, can improve the qualitative representation of the *Arabidopsis* proteome.

## Supplementary Materials

Supplementary materials can be found at http://www.mdpi.com/1422-0067/16/01/0857/s1.
